# A Low-Dimensional Layout of Magnetic Units as Nano-Systems of Combinatorial Logic: Numerical Simulations

**DOI:** 10.3390/ma14112974

**Published:** 2021-05-31

**Authors:** Dominika Kuźma, Paweł Kowalczyk, Krzysztof Cpałka, Łukasz Laskowski

**Affiliations:** 1 Institute of Nuclear Physics Polish Academy of Sciences, PL-31342 Krakow, Poland; Dominika.Kuzma@ifj.edu.pl; 2 Department of Animal Nutrition, The Kielanowski Institute of Animal Physiology and Nutrition, Polish Academy of Sciences, PL-05110 Jabłonna, Poland; p.kowalczyk@ifzz.pl; 3 Institute of Computational Intelligence, Czestochowa University of Technology, 42-200 Czestochowa, Poland; krzysztof.cpalka@pcz.pl

**Keywords:** magnetic particles, numerical simulations, combinatorial logic systems, nanoelectronics, macrospins, micromagnetic simulation, multistage switching

## Abstract

Nanotechnology has opened numerous ways for physically realizing very sophisticated nanodevices that can be fabricated exclusively using molecular engineering methods. However, the synthesis procedures that lead to the production of nanodevices are usually complicated and time consuming. For this reason, the destination materials should be well designed. Therefore, numerical simulations can be invaluable. In this work, we present numerical simulations of the magnetic behaviour of magnetic units shaped into nanometric strips as a low dimensional layout that can be used as nano-systems of combinatorial logic. We showed that magnetic layouts that contain fewer than 16 magnetic units can take on a specific configuration as a response to the input magnetic field. Such configuration can be treated as an output binary word. The layouts that contained various numbers of magnetic units showed different switching characteristics (utterly different order of inverting of strips’ magnetic moments), thus creating numerous combinations of the output binary words in response to the analog magnetic signal. The number of possible output binary words can be increased even more by adding parameters––the system’s initial magnetic configuration. The physical realization of the model presented here can be used as a very simple and yet effective encryption device that is based on nanometric arrays of magnetic units rather than an integrated circuit. The same information, provided by the proposed system, can be utilized for the construction of a nano-sensor for measuring of magnetic field with the possibility of checking also the history of magnetization.

## 1. Introduction

Molecular engineering [1,2], one of the most important tools in nanotechnology, enables to broke the frontiers in the modern technology [3]. Nanotechnology can be considered as some kind of “reversed physics”. For classical physics, we start from the “solving” the materials to find all the properties they have, their structure, and all of the physical laws that apply to them. Once we have these, we can consider some possible applications for the investigated matter. In the nanotechnological approach, we start from considering the most prospective application for some unknown material after which we can try to find some physical and chemical properties that enable it to be used in a manner being considered. In the next step, the molecular structure should be designed in such a way as to imply assumed properties to the resulting material. Next, we should design and execute the synthesis. Having the synthesized material, the classical physical methods can be used to verify the assumptions.

The approach presented above is quite effective, as far as synthesizing the materials for practical applications is concerned. However, It is not an easy process to do that. Both synthesizing a material with assumed properties, as well as designing the correct molecular structure, is extremely difficult in most cases. Considering the latter process, numerical simulations can help significantly. Let us consider the layout of regular magnetic units. When we assume suitably small dimensions of the units, the fabrication of a super-dense memory storage, magnetic nanosensors, molecular neural networks or combinational logic nanocircuit becomes possible [4,5,6,7,8,9,10,11,12,13]. Importantly, the last-mentioned application seems to be promising because such systems can be used in many emerging technologies, such as encryption, encoding, or data compression.

The system can be theoretically fabricated using electrochemical methods [14,15] combined with other nanotechnology tools, which can be selected depending on the assumed geometry, which should be thoroughly thought out because the magnetic behavior of low-dimensional nano layouts is not always obvious. Such a system’s magnetic response to the magnetic field that is applied depends strongly on the number of magnetic units in the whole system. This factor is crucial because it enables the binary encoding of the analog input signal. We describe the assumed operating of the nanometric combinatorial logic system further in the text.

## 2. Materials and Methods

**Figure 1:**
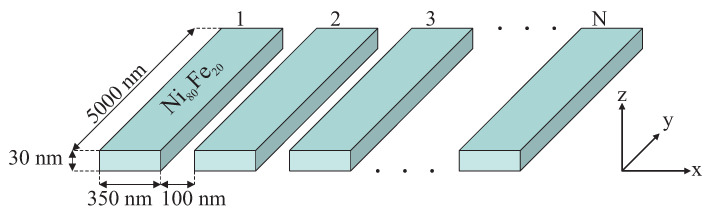
A schematic illustration of the model that was used to approximate the finite layout of the magnetic units to be used as a combinational logic nano circuit.

In this study, we considered the properties of a chain layout of magnetic units regarding the number of magnetic particles [16,17]. The model system is composed of magnetic rectangular strips (350 nm wide, 5000 nm long, and 30 nm thick), which were laid in a regular linear layout as is depicted in Figure 1. The distance between the magnetic strips was 100 nm. The material of strips was permalloy Ni${}_{80}$Fe${}_{20}$ with saturation magnetization of 890 kA/m and exchange stiffness parameter of 1.3·10${}^{-11}$ J/m [18,19]. Considering the assumed use of the model as a combinational logic nano circuit, we paid special attention to the switching properties of the material. As we show below, based on the numerical simulations, the magnetic response of the chain of magnetic particles on the applied magnetic field strongly depended on the number of magnets (magnetic units) in the chain and was very irregular. We studied this irregularity with regard to using it in the binary encoding of an analog signal. The simulations that are presented are part of the molecular design of actual nanoelectronic systems and seem to be crucial in this process.

The general magnetic behavior of the system presented in Figure 1 was analyzed in detail in our previous work [20]. The present paper is a continuation of our earlier investigations. Here, we exploit and present the applicative potential of the system in nanoelectronics. For this reason, we describe the system’s switching properties using various numbers of magnetic units in the layout and focus on the features that are important for the binary coding of an analog input signal (the magnetic field that is applied).

A detailed description of the model and details of the simulations were described in our earlier article [20]. In short, we assumed that a magnetic field is applied to the chains in the *Y* direction (parallel to the long axes of the magnetic units). In order to find the equilibrium configuration for each field value, we minimized the magnetic energy using MuMax software [21,22]. As far as the simulations are concerned, we focused on the following numbers of magnets: 5, 7, 8, 10, 15, 16, 30, 45, 60, 91, 151, 200, and infinite. The most promising ones were the numbers fewer than 16, while the higher numbers were treated as references for the discussion. Obviously, we also investigated other numbers of magnets in the layout. However, here, we present only the most interesting and most important cases. It is crucial to highlight that an even or odd number of magnets behaved differently when the number of magnets was close because of the different symmetry with regards to the central point.

The model that is presented is the first approach to an actual device: the layout of permanent magnetic units fabricated at a nanometric scale. Such a system can theoretically be done using a few methods. The first and most obvious method is electrodeposition using a nanolithographic shutter [23,24,25,26]. Considering the current state of the technology, fabricating the geometry presented in Figure 1 does not seem to be a problem. When we consider smaller systems, however, another method should be used. In these cases, rather than a nanolithographic shutter, an ordered porous matrix that deposited on an electrode can be used during the electrodeposition. These methods result in systems of ordered cylinders rather than strips, but after cutting of the properly oriented thin strip using a focused ion beam (FIB), the final geometry would be similar to the one presented here. As a shutter, the porous anodic alumina matrix [27,28,29] can be used to fabricate various systems with strips ranging from 300 nm down to 10 nm wide. Even smaller units can be obtained by using inside ordered porous silica matrices [30], which can be prepared in the form of vertically aligned systems of pores [31,32]. In this case, we can even go as low as 2 nm wide.

It seems to be clear that the physical implementation of a system using magnetic strips is feasible. What is more, the geometry of these systems can be tuned. For this reason, simulations of the magnetic behavior of low-dimensional magnetic layouts seem to be justified for designing and fabricating the nanometric system that are to be used in nanoelectronics.

## 3. Results and Discussion

**Figure 2:**
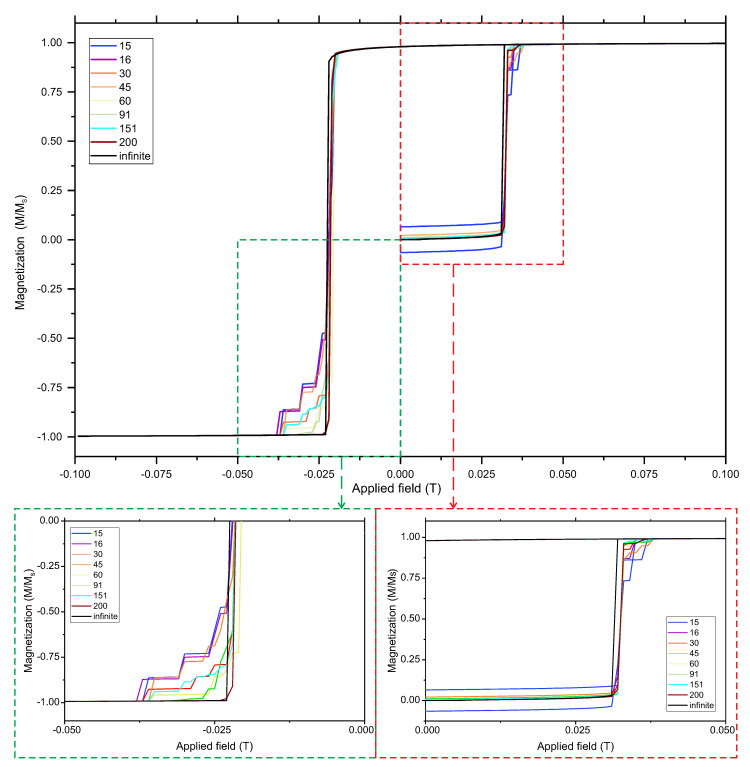
Hysteresis loops for the system with the selected number of magnetic units that were used along with an enlarged view. The second part of the hysteresis loop (field from minimum to maximum) was omitted for the clarity of the picture.

The dependence of a magnet’s behavior on the number of magnets in the layout of a chain is presented in Figure 2. As a starting point, we assumed the antiferromagnetic (AF) arrangement of the magnets. However, after the magnetic field was saturated, the system’s configuration was ferromagnetic (FM) and this was the starting point for decreasing of the magnetic field.

Looking at the plot (Figure 2, one can easily observe that the finitude of the number of magnets in the system strongly influenced the existence of the states that are intermediate between AF and FM, and FM and a reversed ferromagnetic (revFM) state. In this case, there are two distinguished magnets in the system that require attention: the first and the last magnets. What is more, the number of intermediate states and their configuration was strongly dependent on the number of magnets in the chain, the parity of the number of strips in the chain and the initial configuration of the system (AF or FM). This fact is extremely important for the practical application of an actual system as a combinatorial logic element. It can clearly be seen that the steps (intermediate states) become practically invisible for 200 magnetic strips in the chain. Such a chain behaves similar to an infinite chain. In the case of an infinite chain, in turn, the intermediate states do not exist. The reason for this is that none of the magnets is distinguished and the only possibility for reorienting the magnetic field is to flip all of the strips simultaneously. For this reason, only low-dimensional chains of magnetic strips can be considered as a part of the nanoelectronic systems for encoding, encryption and data compression.

**Figure 3:**
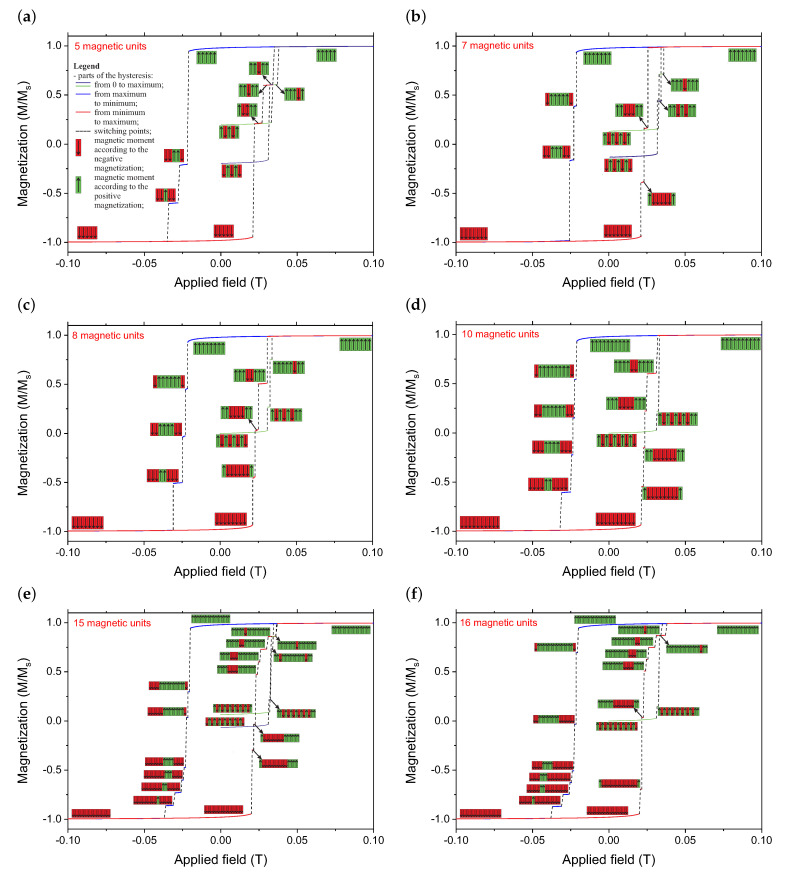
Magnetic hysteresis loops for the system with the selected number of magnets (5—(**a**), 7—(**b**), 8—(**c**), 10—(**d**), 15—(**e**) and 16—(**f**)) along with the magnetic configurations of the magnets as a function of the magnetic field. A magnetic field from 0 to the maximum value, back to the minimum and once again increased to the maximum was applied. The configurations of the systems as a response to the input magnetic field are presented along with the plots. The legend for all of the plots is presented in the first plot (**a**).

In Figure 3, we present the magnetic behavior of the low-dimensional layouts with the selected number of magnets. The starting points for a zero-field are antiferromagnetic configurations. In the case of the odd numbers of magnetic strips in the system, we present both possible AF configurations. We present 5, 7, 8, 10, 15, and 16 strips as examples.

Looking at the selected runs that are presented as examples in Figure 3, we can clearly distinguish the steps in the hysteresis loops for all of the scenarios. Each step corresponds to the system’s specific configuration, which can be written as a system of 0 and 1 values. For avoidance of doubt, each strip is treat as a monodomain ferromagnetic particle (unit). The value “1” corresponds to the magnetization upwards, while the value “0” correspond to the magnetization downward. For total clarity, we juxtaposed all of the system configurations that corresponded to magnetic field ranges and proposed a binary translation of the configuration in Table 1.

**Figure 4:**
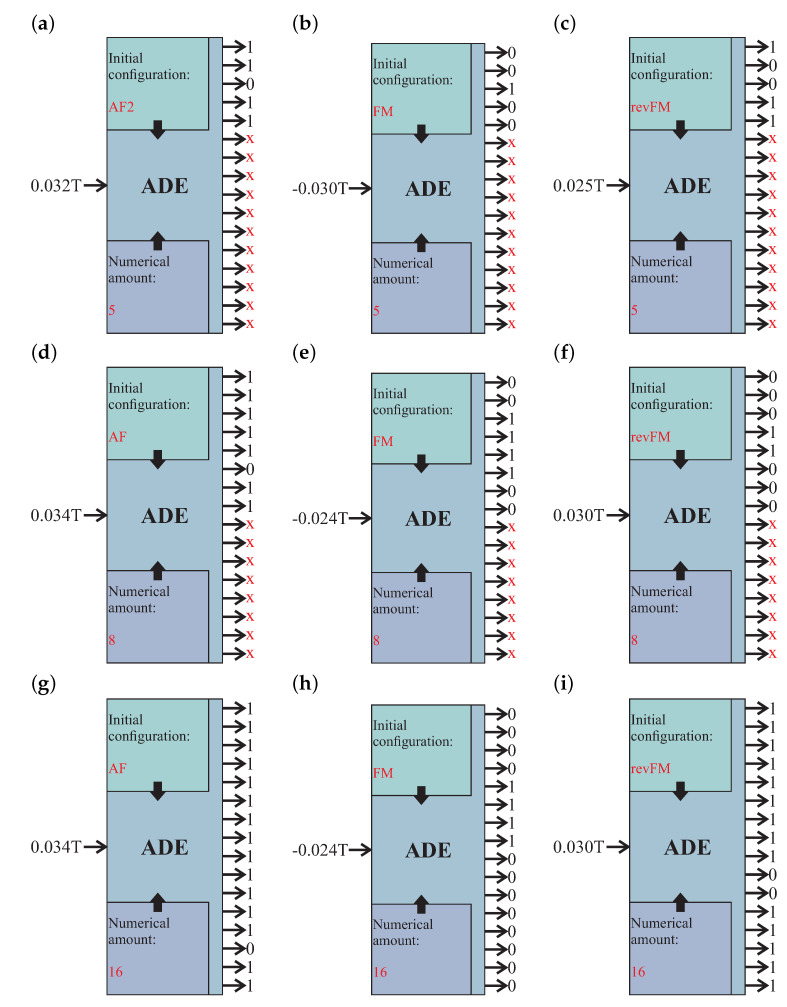
Exemplary ways of coding of an analog signal into a binary code for the proposed analog-to-digital encoder (ADE) based on a finite system of magnets with various numerical amounts of magnetic strips (5—(**a**,**b**,**c**), 8—(**d**,**e**,**f**), and 16—(**g**,**h**,**i**)). The various additional parameters are also presented: the numerical amount of the system and initial magnetic configurations: antiferromagnetic–AF (for the even systems—figures **d** and **g**), AF1 or AF2 (for the odd systems: AF1 means antiferromagnetic with more units antiparallel to the magnetic field, while AF2 means antiferromagnetic with more units parallel to the magnetic field—figure **a**), FM (ferromagnetic, according to the positive value of the magnetic field—figures **b**, **e**, **h**) or revFM (ferromagnetic, antiparallel to the magnetic field—figures **c**, **f** and **i**). The “x” indicates an inactive output.

Thus, each presented system can theoretically be applied as part of a nanoelectronic combinatorial logic system; for a response to some of the applied magnetic fields (analog signal), the system takes a specific configuration that can be interpreted as a binary word (binary output). What is more, the output binary word not only depends on the input signal but also on additional parameters: the number of magnets in the system, as well as its initial configuration. Here, we must mention that the initial configuration is connected with the history of magnetization and can be AF (for even systems), AF1 or AF2 (for odd systems: AF1 means more units according to the negative field, while AF2 means more units according to the positive field–see Figure 3, FM (ferromagnetic, according to the positive value of the magnetic field) or revFM (ferromagnetic, according to the negative value of the field). All of these increase the number of possible binary answers for the system proposed here. The proposed system can be described as a coder coupled with an analog-to-digital converter (ADC). Here, we called it an analog-to-digital encoder (ADE). This type of device can be used as a very simple and effective encryption device that is based on arrays of nanometric magnetic units rather than an integrated circuit. In this case, the proposed system of magnetic strips is the main part of the ADE. Thus, the magnetic field interacting with a magnetic layout causes occupation of some specific configuration corresponding to this field. This configuration can be translated into binary words corresponding to the outputs. We illustrate some exemplary ways of coding for the system in Figure 4.

For clarity, let us analyze the example depicted in Figure 4d. The first ADE parameter is the system’s numerical amount equals eight. In this case, the central part of the system—the nanometric layout of the magnetic units has eight strips, as in Figure 3c. The standard ADE device has 16 assumed outputs. Thus, taking eight magnetic units in the magnetic system, only eight outputs will be active, the next eight will be unavailable (x-marked outputs). The second parameter is the initial magnetic configurations: antiferromagnetic. In this case, we need to increase the magnetic field from zero to the assumed value. We can see that the input magnetic field has a value of 0.034T. With increasing the magnetic field to 0.034T, the magnetic layout takes the configuration seen in Figure 3c and Table 1 for the value of 0.034T and initial run from 0T: 11111011. This configuration is read and provided to the output of ADE.

Another obvious application of such a system can be a nano-sensor of a magnetic field. The binary information about the field and, importantly, about the history of magnetization is provided by the configuration of the proposed layout of magnetic units. The binary coding of the history of magnetization distinguishes our concept device among others proposed in the literature.

## 4. Conclusions

In this study we have presented a numerical model of a low-dimensional layout of magnets that can theoretically be used as a nanoelectronic device to encode an analog signal (magnetic) into a system of binary digits (digital output). The proposed device has a great applicative potential for encryption tasks or data compression. Our research showed that the number of magnets in the layout is crucial for the operation of the proposed device; a relatively small number of units–200–can approximate a continuous system, which is completely useful in the proposed application (however, some other application possibilities can also be found). The systems that contained up to 16 magnets were the most promising. The layouts that contained various numbers of magnets behaved in different ways, which created numerous combinations of the output binary words in response to the analog magnetic signal. The number of possible output binary words can be increased even more by the additional parameter—the system’s initial magnetic configuration. All of this makes the model magnetic system very interesting as far as its potential application in nanoelectronics is concerned, especially as nanodevices for encryption and data compression or nano-sensors of magnetic field.

The next logical step of the research seems to be to attempt to synthesize an actual system and to determine whether the behavior of such a physical layout is well represented by the simulations that are presented here, which is definitely worth doing.

## Figures and Tables

**Table 1:** The proposed method of the binary coding of the input analog magnetic signal by the various finite systems of magnets is presented in Figure 3.

Legend								
The proposed way of reading of units’ states:	Configuration corresponding to binary digit of 1:		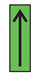	Configuration corresponding to binary digit of 0:		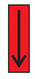	Example: 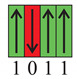	
System of 5 magnetic units								
Initial run from zero field to the maximum value								
Input magnetic field range (T):	0.000–0.033	0.033–0.038	0.038–0.100					
configuration:	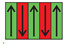	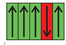	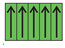					
binary coding:	10101	11101	11111					
Input magnetic field range (T):	0.000–0.031	0.031–0.034	0.034–0.100					
configuration:	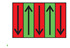	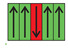	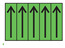					
binary coding:	01010	11011	11111					
The first half of hysteresis: from maximum value to the minimum								
Input magnetic field range (T):	0.100–−0.022	−0.022–−0.028	−0.028–−0.035	−0.035–−0.100				
configuration:	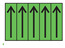	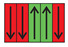	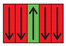	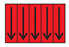				
binary coding:	11111	00110	00100	00000				
The second half of hysteresis: from minimum value to the maximum								
Input magnetic field range (T):	−0.100–0.022	0.022–0.028	0.028–0.035	0.035–0.100				
configuration:	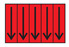	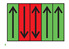	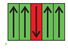	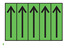				
binary coding:	00000	10011	11011	11111				
System of 7 magnetic units								
Initial run from zero field to the maximum value								
Input magnetic field range (T):	0.000–0.033	0.033–0.037	0.037–0.100					
configuration:	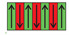	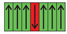	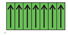					
binary coding:	1010101	1110111	1111111					
Input magnetic field range (T):	0.000–0.032	0.032–0.035	0.035–0.100					
configuration:	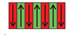	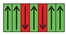	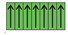					
binary coding:	0101010	1101011	1111111					
The first half of hysteresis: from maximum value to the minimum								
Input magnetic field range (T):	0.100 – −0.022	−0.022 – −0.024	−0.024 – −0.026	−0.026 – −0.100				
configuration:	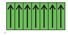	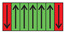	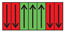	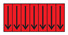				
binary coding:	1111111	0111110	0011100	0000000				
The second half of hysteresis: from minimum value to the maximum								
Input magnetic field range (T):	−0.100–0.022	0.022–0.024	0.024–0.026	0.026–0.100				
configuration:	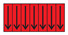	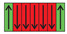	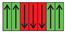	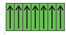				
binary coding:	0000000	1000001	1100011	1111111				
System of 8 magnetic units								
Initial run from zero field to the maximum value								
Input magnetic field range (T):	0.000–0.032	0.032–0.033	0.033–0.035	0.035–0.100				
configuration:	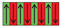	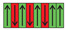	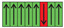	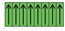				
binary coding:	10101010	10101011	11111011	11111111				
The first half of hysteresis: from maximum value to the minimum								
Input magnetic field range (T):	0.100–−0.022	−0.022–−0.023	−0.023–−0.025	−0.025–−0.031	−0.031–−0.100			
configuration:	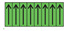	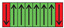	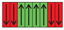	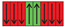	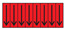			
binary coding:	11111111	01111110	00111100	00011000	00000000			
The second half of hysteresis: from minimum value to the maximum								
Input magnetic field range (T):	−0.100–0.022	0.022–0.023	0.023–0.025	0.025–0.031	0.031–0.100			
configuration:	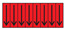	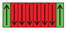	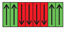	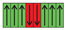	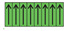			
binary coding:	00000000	10000001	11000011	11100111	11111111			
System of 10 magnetic units								
Initial run from zero field to the maximum value								
Input magnetic field range (T):	0.000–0.032	0.032–0.033	0.033–0.100					
configuration:	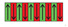	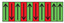	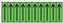					
binary coding:	10101 01010	10101 01011	11111 11111					
The first half of hysteresis: from maximum value to the minimum								
Input magnetic field range (T):	0.100–−0.022	−0.022–−0.023	−0.023–−0.024	−0.024–−0.025	−0.025–−0.032	−0.032–−0.100		
configuration:	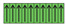	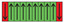	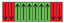	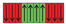	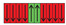	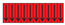		
binary coding:	11111 11111	01111 11110	00111 11100	00011 11000	00001 10000	00000 00000		
The second half of hysteresis: from minimum value to the maximum								
Input magnetic field range (T):	−0.100–0.022	0.022–0.023	0.023–0.024	0.024–0.025	0.025–0.032	0.032–0.100		
configuration:	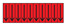	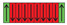	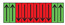	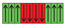	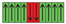	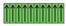		
binary coding:	00000 00000	10000 00001	11000 00011	11100 00111	11110 00001	11111 11111		
System of 15 magnetic units								
Initial run from zero field to the maximum value								
Input magnetic field range (T):	0.000–0.033	0.033–0.037	0.037–0.100					
configuration:	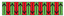	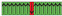	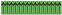					
binary coding:	1010101 01010101	1111111 01111111	1111111 11111111					
Input magnetic field range (T):	0.000–0.032	0.032–0.033	0.033–0.035	0.035–0.100				
configuration:	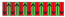	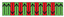	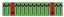	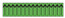				
binary coding:	0101010 10101010	1101010 10101011	1101111 11111011	1111111 11111111				
The first half of hysteresis: from maximum value to the minimum								
Input magnetic field range (T):	0.100–−0.021	−0.021–−0.022	−0.022–−0.023	−0.023–−0.025	−0.025–−0.026	−0.026–−0.031	−0.031–−0.037	−0.037–−0.100
configuration:	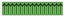	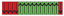	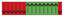	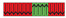	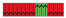	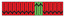	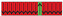	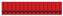
binary coding:	1111111 11111111	0000111 11111110	0000001 11111110	0000000 11110000	0000000 01110000	0000000 01100000	0000000 00100000	0000000 00000000
The second half of hysteresis: from minimum value to the maximum								
Input magnetic field range (T):	−0.100–0.021	0.021–0.022	0.022–0.023	0.023–0.025	0.025–0.026	0.026–0.031	0.031–0.037	0.037–0.100
configuration:	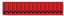	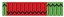	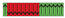	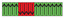	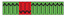	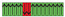	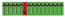	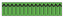
binary coding:	0000000 00000000	1000000 00001111	1000000 00111111	1111000 01111111	1111000 1111111	1111100 11111111	1111101 11111111	1111111 11111111
System of 16 magnetic units								
Initial run from zero field to the maximum value								
Input magnetic field range (T):	0.000–0.032	0.032–0.033	0.033–0.035	0.035–0.100				
configuration:	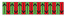	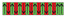	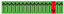	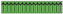				
binary coding:	10101010 10101010	10101010 10101011	11111111 11111011	11111111 11111111				
The first half of hysteresis: from maximum value to the minimum								
Input magnetic field range (T):	0.100–−0.021	−0.021–−0.022	−0.022–−0.023	−0.023–−0.025	−0.025–−0.026	−0.026–−0.031	−0.031–−0.038	−0.038–−0.100
configuration:	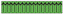	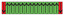	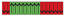	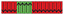	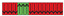	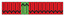	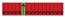	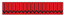
binary coding:	11111111 11111111	01111111 11111110	00111111 11000000	00001111 00000000	00001110 00000000	00000110 00000000	00000100 00000000	00000000 00000000
The second half of hysteresis: from minimum value to the maximum								
Input magnetic field range (T):	−0.100–0.021	0.021–0.022	0.022–0.023	0.023–0.025	0.025–0.026	0.026–0.031	0.031–0.038	0.038–0.100
configuration:	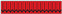	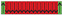	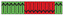	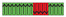	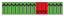	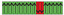	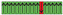	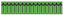
binary coding:	00000000 00000000	10000000 00000001	11111100 00000011	11111111 00001111	11111111 10001111	11111111 10011111	11111111 11011111	11111111 11111111

## Abbreviations

The following abbreviations are used in this manuscript:

FM	Ferromagnetic
AF	Antiferromagnetic
AF1	Antiferromagnetic for odd number of magnetic units in the system: more units antiparallel to the magnetic field
AF2	Antiferromagnetic for odd number of magnetic units in the system: more units parallel to the magnetic field
revFM	reversed ferromagnetic, antiparallel to the magnetic field
ADC	Analogue-to-digital converter
ADE	Analogue-to-digital-encoder
